# Investigation of Biocidal Effect of Microfiltration Membranes Impregnated with Silver Nanoparticles by Sputtering Technique

**DOI:** 10.3390/polym12081686

**Published:** 2020-07-29

**Authors:** Aline M. F. Linhares, Cristiano P. Borges, Fabiana V. Fonseca

**Affiliations:** 1 School of Chemistry, Federal University of Rio de Janeiro, Horacio Macedo Av, 2030, Technology Center, I-124, University City, Rio de Janeiro 21941-909, Brazil; fabiana@eq.ufrj.br; 2 Chemical Engineering Program, COPPE, Federal University of Rio de Janeiro, Horacio Macedo Av, 2030, Technology Center, G-115, University City, Rio de Janeiro 21941-450, Brazil; cristiano@peq.coppe.ufrj.br

**Keywords:** silver nanoparticles, microfiltration, membranes, biofouling, sputtering

## Abstract

Silver nanoparticles were loaded in microfiltration membranes by sputtering technique for the development of biocidal properties and biofouling resistance. This technology allows good adhesion between silver nanoparticles and the membranes, and fast deposition rate. The microfiltration membranes (15 wt.% polyethersulfone and 7.5 wt.% polyvinylpyrrolidone in *N*,*N*-dimethylacetamide) were prepared by phase inversion method, and silver nanoparticles were deposited on their surface by the physical technique of vapor deposition in a sputtering chamber. The membranes were characterized by Field Emission Scanning Electron Microscopy, and the presence of silver was investigated by Energy-Dispersive Spectroscopy and X-ray Diffraction. Experiments of silver leaching were carried out through immersion and filtration tests. After 10 months of immersion in water, the membranes still presented ~90% of the initial silver, which confirms the efficiency of the sputtering technique. Moreover, convective experiments indicated that 98.8% of silver remained in the membrane after 24 h of operation. Biocidal analyses (disc diffusion method and biofouling resistance) were performed against *Pseudomonas aeruginosa* and confirmed the antibacterial activity of these membranes with 0.6 and 0.7 log reduction of viable planktonic and sessile cells, respectively. These results indicate the great potential of these new membranes to reduce biofouling effects.

## 1. Introduction

Microfiltration process (MF) has many consolidated advantages over conventional separation processes, mainly due to its ease of operation and low energy consumption, being widely used for disinfecting water. An evaluation of the global MF membrane market has indicated an expected annual growth rate of 9.0% from 2018 to 2023 [1]. However, membranes’ fouling and biofouling are major drawbacks, reducing permeate flux and increasing operational costs. Usually, authors consider that biofouling can be one of the most difficult deposits on membranes to eliminate, highlighting the fast microbes growth, even at low nutrient concentrations [2,3,4].

To minimize such problems, one approach is to modify the properties of the surface of the membrane. For instance, silver nanoparticles (AgNps) are known for their bactericidal characteristics, which can work against microorganisms growth and, consequently, against biofouling on membranes [2,5,6,7]. Besides water disinfection, microfiltration membranes with biofouling resistance can be used for several applications such as membrane bioreactors and pretreatment for nanofiltration and reverse osmosis.

The biocidal mechanism of silver nanoparticles is not completely understood. However, the most accepted hypothesis is that silver ions interact with thiol groups in proteins, resulting in inactivation of enzymes and leading to the production of reactive oxygen species (ROS). Another important mechanism is the adhesion of AgNps to the surface of microorganisms, which alters the exchange of nutrients, salts, and water. DNA damage, resulting from AgNps penetrating the bacterial cell, can be highlighted as well [8,9,10,11,12,13,14].

On the other hand, the use of AgNps membranes for the disinfection of water should consider any possibility of risks to human health. It is known that levels of silver up to 0.1 mg L^−1^ can be tolerated. However, long-term exposures to silver at high concentrations can generate skin darkening (argyria) [15,16,17]. Although the existing standards protect consumers from nanoproducts, there are still gaps in the assessment of risks for humans and some aspects need to be optimized, such as limits of toxicity, dose, and concentration to aquatic organisms and humans [14,16,18,19].

Silver nanoparticles may be synthesized by many methods and chemical reduction is the most commonly used. However, these routes present several reaction steps, and their residual solvents depict environmental concerns. Furthermore, AgNps tend to aggregate during their preparation in solution, thus the use of coating agents is necessary, which may reduce their antibacterial activity [2,20].

Physical techniques of vapor deposition, such as sputtering, allow the modeling of the size and distribution of the particles, as well as the high deposition rate and the good adhesion between AgNps and membranes. This technique uses high-energy ions to carry atoms from a target, which acts as a cathode, and deposit them onto a substrate. The last one acts as the anode in a sputtering chamber filled with inert gas. The releasing of Ag plasma ions moves with high kinetic energy and condenses as nanoparticles on the supporting material [21,22].

Previous studies reported concerning the inhibition of microorganism growth after contact between bacteria suspension and polymeric membranes impregnated with nanoparticles have been published. Among them, many reported the biocidal effect of AgNps membranes, but for short periods of time and with a continuous release of AgNps. This issue reduces the bactericidal properties of the membrane over time and can cause overestimations of the overall efficiency of the membrane [7,20,23]. For example, Dong et al. (2017) [7] showed 100% of mortality of *Escherichia coli* and *Bacillus subtilis* suspensions. Furthermore, Dong et al. (2019) [24] observed a significant suppression of *Serratia marcescens* using membranes loaded with AgNps. However, it should be considered that these studies evaluated the silver loss, either through membrane immersion or filtration process, only in short-term periods. On the other hand, Park et al. (2016) [20] verified the strong antibacterial activity against *E. coli*, *Pseudomonas aeruginosa*, and *Staphylococcus aureus*, even though the membrane was expected to last no longer than 97 days, while the estimation of Liu et al. (2015) [23] was of 340 days. Thus, nanoparticles leaching still poses a challenge to overcome, which suggests the development of new techniques to solve this problem [25,26].

In this work, silver nanoparticles were loaded on the surface of polymeric membranes by sputtering technique, aiming at the development of membranes with biocidal properties that would be resistant to biological fouling and capable of being used in the disinfection of water.

Silver leaching was extensively investigated through immersion and convective experiments.

## 2. Materials and Methods

### 2.1. Materials

Polyethersulfone (PES, MW 58 kDa) and polyvinylpyrrolidone (PVP, MW 360 kDa) were purchased from Basf, Ludwigshafen am Rhein, Germany and Sigma-Aldrich, St. Louis, MI, USA, respectively. The common solvent for both polymers was *N*, *N*-dimethylacetamide (DMAc, 99.5%), purchased from Tedia, Fairfield, Ohio, EUA. For microbiological experiments, deionized water was supplied by a Milli-Q apparatus (Merck KGaA, Darmstadt, Germany). Yeast extract, meat peptone, and agar were purchased from Kasvi, São José do Pinhais, Brazil. Magnesium sulfate, potassium phosphate monobasic, potassium phosphate dibasic, and glycerol were purchased from Vetec, Duque de Caxias, Brazil. These reagents were used as culture medium. PES and PVP were dried at 60 °C overnight before being used and the other reagents were used as received.

### 2.2. Microfiltration Membrane

The microfiltration membranes were prepared by phase inversion method [27]. Briefly, a polymer solution with 15 wt.% PES and 7.5 wt.% PVP in DMAc was prepared by continuous stirring at room temperature. After degassing overnight, the solution was cast onto a glass plate with a 200 μm thick casting knife and exposed to the ambient atmosphere (60% RH) for 100 s prior to immersion in a precipitation bath composed of 80 wt.% DMAc and 20 wt.% deionized water. After complete precipitation (~15 min), the resulting membrane was immersed three times in a deionized water bath for 2 h to remove any residual solvent.

### 2.3. Silver Nanoparticles Deposition

Silver nanoparticles were directly deposited on the surface of the microfiltration membrane (4.7 cm in diameter) by sputtering (Quorum Q150R ES, Quorum Technologies, Laughton, UK) at room temperature and under a low-pressure argon atmosphere (0.1 Pa). A silver target (99.9% Ag, 57 mm in diameter and 0.1 mm thick, Sigma Aldrich, St. Louis, MI, USA) located in the center of the vacuum chamber acts as a cathode and the MF membranes are used as a substrate for deposition. The Ag plasma ions move with high kinetic energy to be condensate as nanoparticles on the surface of the membrane.

The MF-AgNps membranes were obtained with 15 mA (power = 6.0 W) and 50 mA (power = 26.0 W) of sputtering current and 15 and 120 s of deposition time, respectively, as described elsewhere [28]. These membranes are referred to as MF-15mA-15s and MF-50mA-120s, respectively. The chosen conditions aimed to investigate the biocidal properties of the membranes with different content of silver (8.22 and 317.78 mg m^−2^ on MF-15mA-15s and MF-50mA-120s, respectively). For comparison purposes, a membrane without sputtering treatment was used as a control (MF-membrane).

### 2.4. MF-AgNps Membrane Characterization

The surfaces of MF-AgNps membranes were verified by Field Emission Scanning Electron Microscopy (FESEM, ZEISS Auriga 40, Ulm, Germany) and the presence of silver was evaluated by Energy-Dispersive Spectroscopy (EDS). Samples were coated with carbon using a metallizer (Quorum Emitech K550, Quorum Technologies, Kent, UK) for FESEM and with gold for EDS.

X-ray Diffraction (XRD, Rigaku Miniflex II, Rigaku, The Woodlands, TX, USA) was also used to verify the silver nanoparticles loaded by the sputtering technique and to assess their crystallinity. The data were collected in the 2-theta range of 5° to 90° and scanning speed of 0.05 s^−1^. The average size was calculated using the Debye-Scherrer formula, presented in Equation (1):

(1) $$D_{p}=\frac{K\lambda }{\beta_{1/2}cos\theta }$$

where *D_p_* is the crystallite size, *K* is a numerical factor referred to as the crystallite-shape factor (*K* = 0.9 is a good approximation), *λ* is the wavelength of the X-rays (*λ* = 1.5418 Å), *β*_1/2_ is the full-width at half-maximum of the X-ray diffraction peak in radians, and *θ* is the diffraction angle.

Fourier Transform Infrared Spectroscopy (FTIR) analysis was conducted on Agilent Cary 630, Santa Clara, Califórnia, EUA (wavenumber range 500 to 4000 cm^−1^; 32 scans at a resolution of 4 cm^−1^).

Silver leaching from MF-AgNps membranes was evaluated through their immersion in water and their performance in convective experiments.

The immersion tests were performed following an established protocol that has been extensively reported in the literature [2,20,23,28,29]. The MF-AgNps membrane coupons (4.7 cm in diameter) were soaked in 50 mL of deionized water at 25 °C and stirred at 60 rpm in a shaker. Water samples were collected after 1, 4, and 24 h of immersion. Long duration tests were also conducted over 1 and 10 months of immersion without intermediate sampling. All samples were acidified to pH 2.0 with HNO_3_ (2% v/v), and then analyzed by inductively coupled plasma-atomic emission spectrometry (ICP-AES) to quantify the amount of dissolved Ag.

Silver content in the membranes and silver loss percentage were also quantified by the digestion of other coupons of the MF-AgNps membranes that were immersed into HNO_3_ (10% v/v) and sonicated for 3 h to ensure that all the AgNps present in the membrane were leached to solution. The total amount of Ag deposited onto the MF-AgNps membranes was assessed by ICP-AES [5,20].

**Figure 1:**
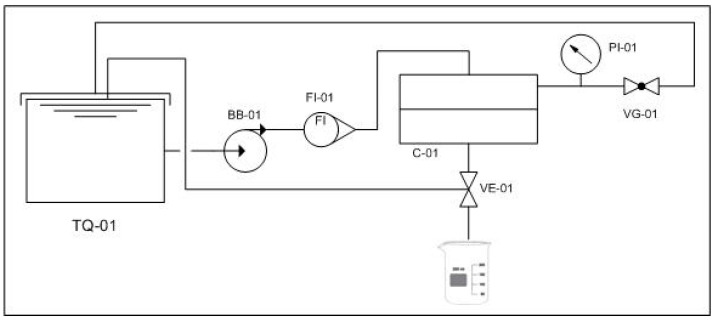
Cross-flow membrane system for silver leaching experiments.

The convective experiments were performed in a cross-flow membrane system, as illustrated in Figure 1, which had an effective membrane area of 45 cm^2^.

The membrane system was designed to work with or without recirculation of permeate and retentate to the feed tank (TQ-01). The system consisted of a pump (BB-01) that transfers the solution from the feed tank to the permeation cell (C-01) with a flowrate meter (FI-01) between them. The permeate stream could be collected or recirculated by switching the valve (VE-01), and the retentate stream was continuously recirculated. The system’s pressure was measured by a control pressure gauge (PI-01) and adjusted through a valve (VG-01).

The silver leaching experiments were conducted with the operational pressure set to 1.5 bar and a flow rate of 40 L h^−1^. 

Additionally, the water flux was calculated using Equation (2) at specific transmembrane pressure, and the permeability was acquired by the slope of the curve *J_p_* × pressure (i.e., 0.5 to 1.5 bar).

(2) $$J_{p}=\frac{V}{A\times t}$$

where *J_p_* is the water flux (L h^−1^m^−2^), *V* is the permeate volume (L), *A* is the effective membrane area (m⁻^2^), and *t* is the filtration time (h).

*P. aeruginosa* suspension (10^8^ UFC mL^−1^) was used as an organism probe to evaluate the rejection capacity (Equation (3)) of each membrane at 1.5 bar.

(3) $$R\;\left(\%\right)=1-\frac{C_{p}}{C_{f}}\times 100$$

where *C_p_* and *C_f_* is the final viable *P. aeruginosa* concentration of the permeate stream and feed solution, respectively.

### 2.5. Antibacterial Activity Tests

For the antibacterial activity tests, Gram-negative bacterium *P. aeruginosa* was selected as the model organism. *P. aeruginosa* is considered the paradigm organism for microbial biofilm studies due to its ability to quickly adhere to many different surfaces, its high reproduction rate, and its significance as a pathogen [25,30,31].

*P. aeruginosa* cells were inoculated into liquid culture medium and incubated with continuous stirring at 200 rpm overnight at 30 °C. This cell suspension served as a bacterial stock solution, which was further diluted to a specific concentration for each test.

The liquid culture medium was prepared with 5.0 g L^−1^ yeast extract, 5.0 g L^−1^ meat peptone, 0.2 g L^−1^ magnesium sulfate, 7.0 g L^−1^ potassium phosphate dibasic, 3.0 g L^−1^ potassium phosphate monobasic, and 30 g L^−1^ glycerol. The solid culture medium was produced from the same solution with the addition of 18 g L^−1^ agar.

#### 2.5.1. The Disc Diffusion Method

The antibacterial activity of the MF-AgNps membranes was first investigated by a disc diffusion method against *P. aeruginosa*. Membrane coupons (17 mm in diameter) were previously sterilized by ultraviolet irradiation for 15 min. Then, the upper surface of the membranes, which holds the silver nanoparticles, was put in contact with the agar plates containing *P. aeruginosa* bacteria at a concentration of 10^6^ colony forming units per mL (CFU mL^−1^).

After incubation at 30 °C for 24, 48, and 72 h, the presence of inhibition zones was monitored and recorded by a digital camera. This inhibition ring, without microbial growth, served as an indicator of antibacterial activity. Furthermore, MF membrane (without AgNps) and an agar plate without membrane were also observed as control samples. All tests were made in triplicate.

#### 2.5.2. The Biofouling Resistance Tests

The biofouling resistance test was performed to evaluate the activity of AgNps in the prevention of bacterial adhesion on the membrane surface. MF-AgNps membrane coupons (1 cm^2^) were immersed into 10^7^ CFU mL^−1^ *P. aeruginosa* suspensions and incubated for 24 h at 30 °C and 200 rpm stirring. Samples with MF membrane coupons and without membranes were also investigated as controls. All tests were made in triplicate. 

After incubation, the planktonic cells in the supernatant and the sessile cells in the biofilm were counted. For total planktonic cells, the optical density at 600 nm (Shimatsu Mini 1240, Mumbai, India) was monitored and the bacterial concentration was determined. For viable planktonic cells, tenfold dilutions were spread onto agar plates and incubated overnight, and viable bacterial colonies were counted on the following day [5,31].

To measure the number of cells attached to the surface of the membrane (sessile cells), the coupon was rinsed with 20 mL of normal saline (0.9 wt.%) to ensure the removal of unattached cells. Then, the membrane coupon was placed in 15 mL of liquid culture medium and vortexed on the highest setting for 120 s in order to cause biofilm disruption. This supernatant was analyzed through optical density at 600 nm for total sessile cells. For the quantification of viable sessile cells, tenfold dilutions were spread onto agar plates, incubated overnight, and the CFU on the plates were counted on the following day.

The Log Reduction and the bacterial viability were calculated using Equations (4) and (5), respectively [20,32]:

(4) $$Log\;Reduction=log_{10}\frac{N_{0}}{N}$$

(5) $$\;Bacterial\;Viability\;\left(\%\right)=\frac{N}{N_{0}}\times 100\;$$

where *N* is the number of viable cells in contact with MF-AgNps membranes and *N_0_* is the number of viable cells in contact with MF membrane (control—without AgNps).

In order to investigate the occurrence of biofouling in the MF membrane and MF-50mA-120s, a filtration experiment was carried out in a cross-flow membrane system, as indicated in Figure 1. The tests were conducted with recirculation of both permeate and retentate streams at 1.5 bar, with a flow rate of feed of 40 L h^−1^. *P. aeruginosa* suspension of 10^8^ CFU mL^−1^ was prepared and placed in the feed tank, and after 4.5 h of permeation, the viable sessile cells were quantified as described before.

## 3. Results and Discussion

### 3.1. Membrane Characterization

**Figure 2:**
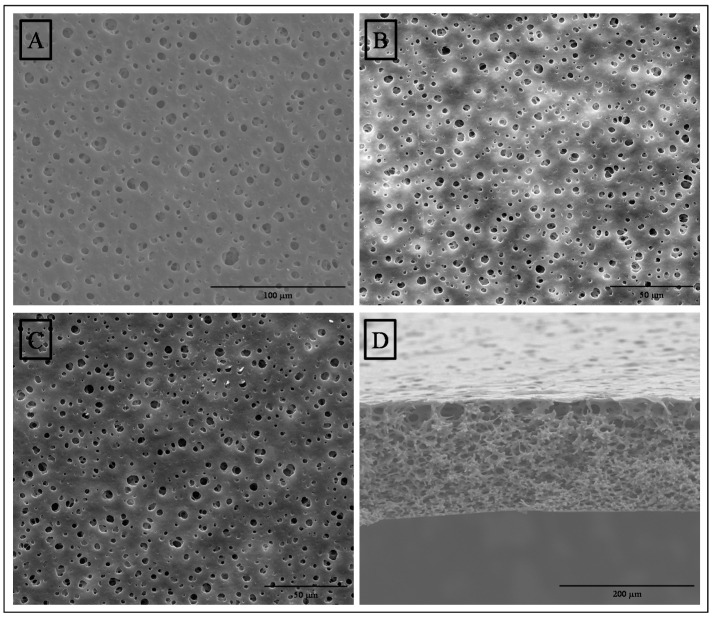
Field emission scanning electron microscopy (FESEM) photomicrographs of the surfaces (1000×) of (**A**) MF-membrane, (**B**) MF-15mA-15s, (**C**) MF-50mA-120s, and of (**D**) cross section of MF membrane (500×).

Figure 2 presents the FESEM photomicrographs of the upper surface and cross section of MF membrane (A and D) and the surfaces of MF-15mA-15s (B) and MF-50mA-120s (C). No significant difference in the surface pores of MF membrane and membranes loaded with silver nanoparticles was observed. The cross section of MF membrane showed a sponge-like morphology with interconnected pores. 

**Figure 3:**
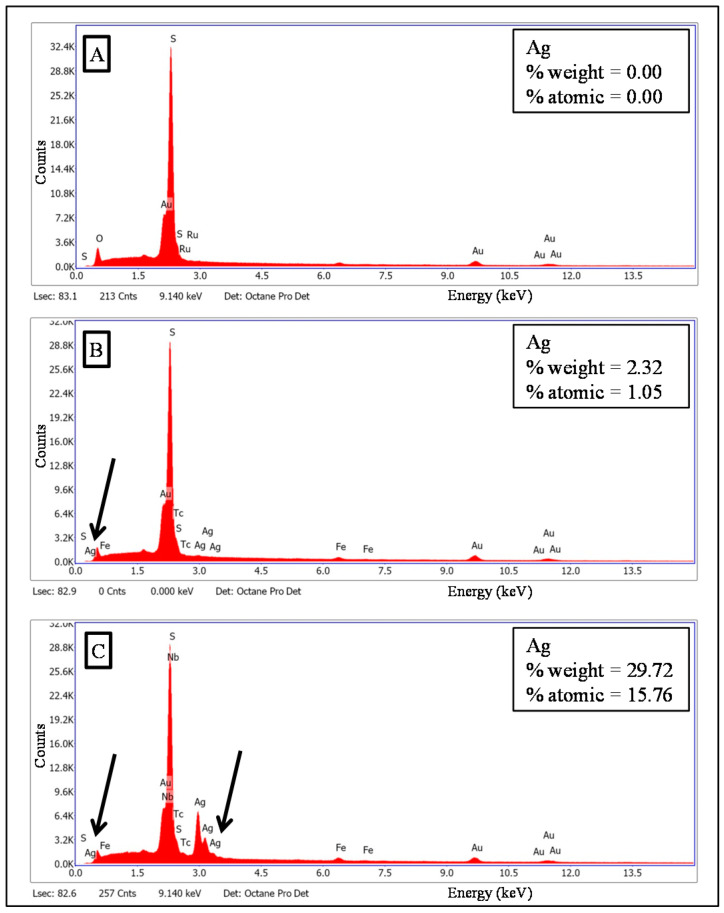
The energy dispersive X-ray (EDS) spectra of membranes (**A**) MF membrane, (**B**) MF-15mA-15s, and (**C**) MF-50mA-120s.

**Figure 4:**
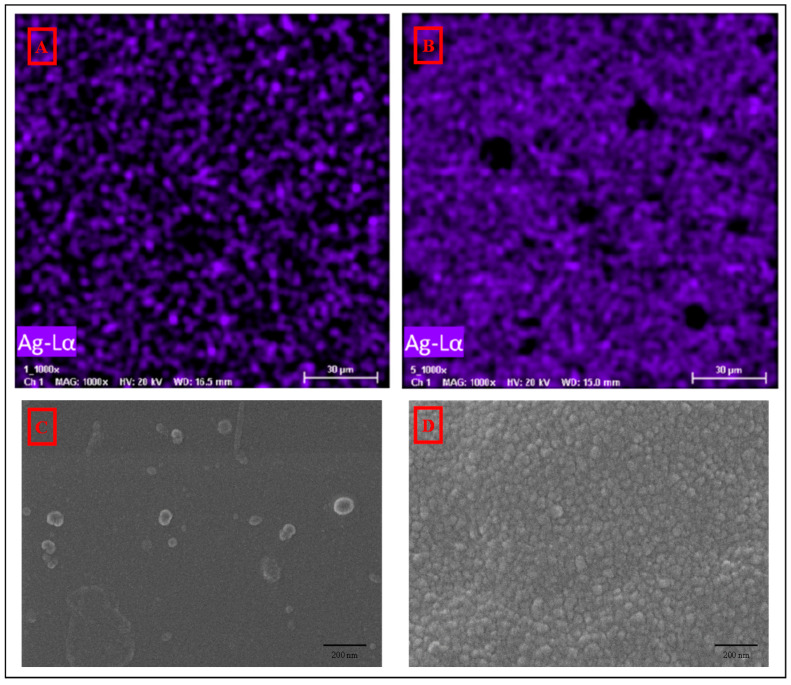
EDS mapping for silver element from (**A**) MF-15mA-15s and (**B**) MF-50mA-120s, and FESEM photomicrographs of the surface of the membranes (100,000×) of (**C**) MF-15mA-15s and (**D**) MF-50mA-120s.

The EDS spectra of the membranes’ surface are portrayed in Figure 3, where the presence of silver element is indicated by black arrows. In addition, Figure 4 shows their EDS mapping and FESEM images. FESEM images exhibit spherical particles on the surfaces of MF-15mA-15s (C) and MF-50mA-120s (D). These spherical particles present average diameters of 88 and 50 nm for MF-15mA-15s and MF-50mA-120s, respectively. The EDS mapping revealed uniform distribution of silver element and, as expected, a larger amount of AgNps in MF-50mA-120s, corroborating what was observed in FESEM images. These results suggest that the increases in sputtering time and sputtering current reduce the diameter of silver particles, which might be related to a higher nucleation rate for nanoparticle growth at higher sputtering current, and confirm that the silver nanoparticles were successfully impregnated on the surface of the MF membranes.

**Figure 5:**
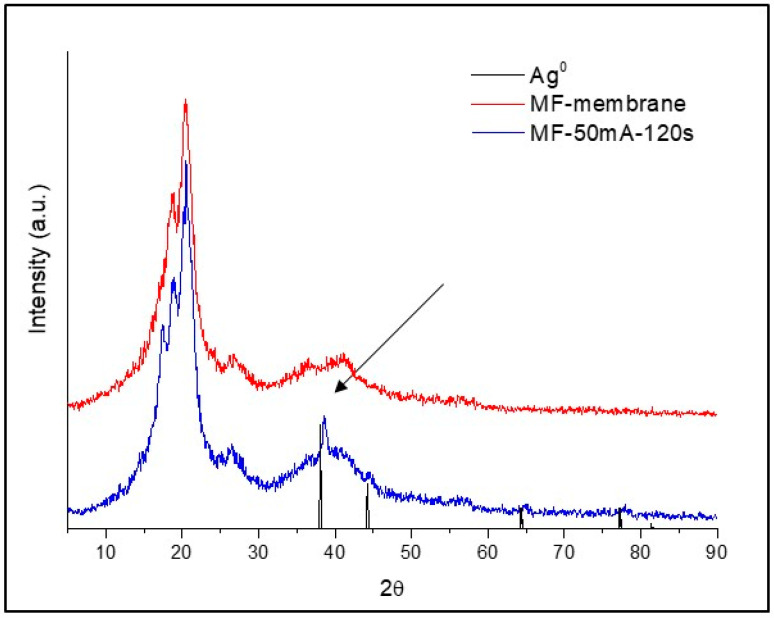
XRD patterns of Ag^0^, MF membrane, and MF-50mA-120s.

X-ray diffraction patterns are shown in Figure 5 from 5° to 90°. The MF membrane exhibited a broad peak that corresponds to the amorphous structure of PES. For MF-50mA-120s membrane, a sharp peak is observed at 2 *θ* = 38°, which is attributed to the crystallinity of silver (black arrow) and represents (111) Bragg’s reflections of face-centered cubic (fcc) structure [33]. These observations are in agreement with values of silver nanoparticles reported in the literature [2,6,33,34,35]. For the MF-15mA-15s membrane, there was no observed peak related to crystalline domains, which may be attributed to the low amount of silver deposited in these sputtering conditions. 

The broadening of peaks in the X-ray diffraction pattern can be related to the particle size [33]; thus, the average size of 17.7 nm was estimated using Scherrer’s equation for the silver nanoparticles in MF-50mA-120s.

The synthesis of nanoparticles by sputtering deposition techniques and their mechanisms of nucleation and growth were investigated by other studies for several metals such as silver [36,37], copper [38], cobalt [39], niobium [40], and palladium [41]. However, the detailed description of these mechanisms is very complex [39,42].

It has been also described that, after long periods (e.g., more than 6 min) of plasma treatment, the surface of PES membranes can be damaged, for instance, the molecular bonds C-C and C-H can be cleaved by argon plasma. On the other hand, the application of short periods of time seams not affect the polymer chains of the membranes [43,44]. Therefore, in this current work, the FTIR spectra (Figure S1 in Supplementary Materials) of the membranes, before and after the impregnation of the nanoparticles, revealed no significant effects of the sputtering technique. The aromatic C-H stretches at 3094 and 3062 cm^−1^ (Figure S1B), and aromatic C=C stretches at 1574 and 1481 cm^−1^ (Figure S1C) remained the same for both membranes.

The water permeability of MF membrane and membranes impregnated with silver nanoparticles are presented in Table 1. The results indicate that there was no significant difference in water permeability between MF membrane and modified membranes (MF-15mA-15s and MF-50mA-120s). This result is in agreement with reported works for different membranes characteristics and AgNps synthesis techniques [7,23,24,45]. 

These membranes present large pore sizes, and they are fabricated with hydrophilic polymers (PES and PVP). Such polymers can input high water flux to the membrane, especially on phase inversion technique, where part of the additive, i.e., PVP, can be entrapped in the membrane matrix, as reported in the literature [46,47,48]. 

The PES membrane contains sulfone and ether groups alternated between aromatic rings [44,49]. The FTIR spectra show characteristic bands of PES: (1) 3094 and 3062 cm^−1^ due to aromatic C–H stretch (Figure S1B) [46,49,50]; (2) 1574 and 1481 cm^−1^ due to aromatic C=C asymmetric stretch (Figure S1C) [44,50]; and (3) 1320 and 1296 cm^−1^ resulting from the anti-symmetric O=S=O stretch of the sulfone group (Figure S1D) [44,49,50]. Moreover, the FTIR spectra shows the presence of PVP with a characteristic band at 1650 cm^−1^ due to carbonyl group (Figure S1E) [46,47,50].

### 3.2. AgNps Releasing Test

**Figure 6:**
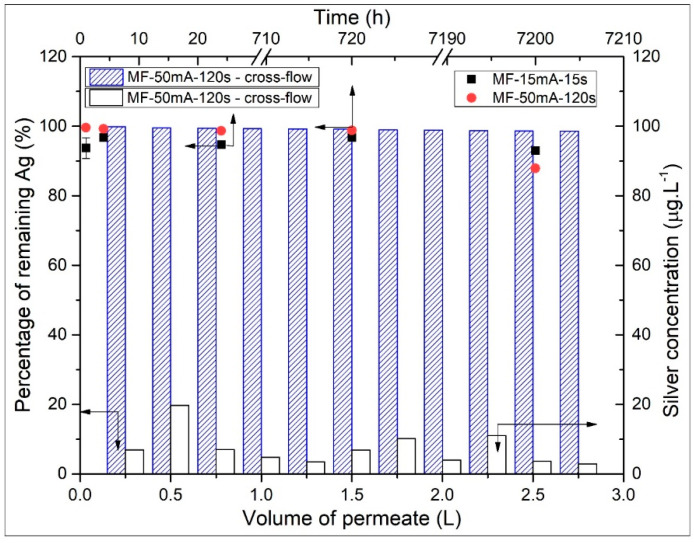
Percentage of the remaining Ag on MF membranes (**left axis**) over time (**upper axis**) in immersion test (**black square**) Ag loaded with 15 mA and 15 s of sputtering; (**red circle**) Ag loaded with 50 mA and 120 s of sputtering). Conditions: diameter of membrane = 4.7 cm, volume of deionized water = 50 mL, 60 rpm. Percentage of remaining Ag (**blue-dashed column**, **left axis**) and silver concentration leaching (**white column**, **right axis**) per volume of permeate (**lower axis**) in cross-flow experiment. Conditions: pressure = 1.5 bar, flow rate = 40 L h^−1^, membrane area = 45 cm^2^.

In order to investigate the stability of silver nanoparticles loaded on MF-AgNps membranes, the concentration of silver leaching was also determined by immersion and filtration experiments (Figure 6). The percentage of the remaining Ag on the modified membranes was calculated based on the total amount of AgNps initially deposited on the surface of the membranes, corresponding to 8.22 and 317.78 mg m^−2^ on MF-15mA-15s and MF-50mA-120s, respectively.

During the first hour of immersion, the MF-15mA-15s membrane lost 6.32 ± 2.98% silver content; however, considering the total period of 10 months (7200 h), a loss of 7.02% was observed, indicating that the biggest part of silver release occurs at the beginning of immersion in the water bath. This fast decrease in the release of silver is qualitatively similar to other studies [7,20,24,51] and may be explained by probable unattached AgNps on the surface of the membrane.

Besides the percentage of silver releasing from the MF-15mA-15s membrane, its concentration in water after 10 months was 20.0 µg L^−1^, which is lower than the silver maximum contaminants limit of 100 µg L^−1^ described by the World Health Organization Guideline for Drinking Water [17] and the U.S. Environmental Protection Agency (USEPA).

Not only did the MF-15mA-15s membrane present a feasible characteristic on the entrapment of silver nanoparticles, but also the MF-50mA-120s showed 1.34 ± 0.13% of silver loss after 24 h of immersion. Furthermore, after 1 month of immersion, the silver concentration in water was similar to the one after 24 h of immersion (0.1 mg L^−1^), which indicates the same trend of silver releasing.

After 10 months of immersion, there was still approximately 93.0 and 87.9% of impregnated silver on MF-15mA-15s (Figure 6, black square) and MF-50mA-120s (Figure 6, red circle), respectively.

Figure 6 also shows the results of silver leaching from MF-50mA-120s membrane during the cross-flow experiment. After 2.75 L of water permeation, there was still 98.5% of silver impregnated on the membrane surface (Figure 6, blue-dashed column), and the concentration of silver leached for each 0.25 L of permeate was lower than 100 µg L^−1^ (Figure 6, white column). 

In addition, a test with recirculation of both permeate and retentate streams (full-recycle setup) was also performed during 24 h. The concentration of silver leached (in the feed tank) was 8.4 µg L^−1^ at the end of the experiment, which indicates that 98.8% of the initial silver impregnated on MF-50mA-120s remained on its surface after permeation. The water permeation test corroborates the silver loss after 24 h of immersion in the water bath and indicates that, even at a flowrate of 40 L h^−1^, the MF-50mA-120s membrane showed a small loss of silver. The results of silver leaching in both experiments indicate that the sputtering technique is effective for impregnating and entrapping silver nanoparticles on the surface of membranes. This finding is important for the maintenance of the membrane’s biocidal performance and for the minimization of silver leaching to the environment. 

Evaluation of silver loss in previous studies of membranes impregnated with silver nanoparticles by chemical reduction method showed percentages of remaining silver of 99.38% and 98.75% after 24 h and six days of immersion, respectively [7]. Furthermore, a percentage of 97.0% was obtained after a cross-flow experiment [24]. However, in these studies, the immersion test was conducted for short periods and under reduced flowrate (cross-flow experiment) in comparison with this work. In addition, the chemical reduction method presents some disadvantages as several steps production and chemical reagents, the use of stabilizer agents and the residual solvents.

Another important fact is a continuous silver loss observed in other studies with physical methods and green synthesis [20,23]. In these cases, the authors indicated that the lifespans of their membranes were 97 and 340 days according to their silver leaching rate. 

In fact, this current work highlights the evaluation of silver loss for long-term immersion and cross-flow experiments for membranes loaded with silver nanoparticles by a one-step production method with no residual reagents. 

### 3.3. Antibacterial Activity Tests

#### 3.3.1. The Disc Diffusion Method

**Figure 7:**
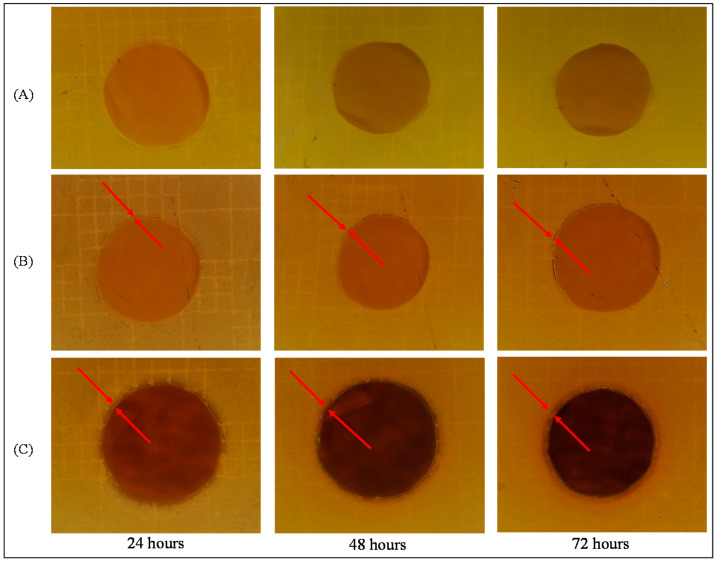
Diffusion disc method against *Pseudomonas aeruginosa* of (**A**) MF membrane (control sample), (**B**) MF-15mA-15s, and (**C**) MF-50mA-120s. Inhibition zone are indicated by red arrows.

As illustrated by the disk tests (see Figure 7), MF-membrane (A) had no significant effect on the growth of *P. aeruginosa*, while MF-AgNps membranes showed a clear area with no evidence of bacterial growth. An inhibition zone around the membranes of 0.5 and 0.8 mm was observed for MF-15mA-15s (B) and MF-50mA-120s (C), respectively. These results are similar to many reported works, which also found the inhibition zone around the substrate containing AgNps, and demonstrate that this antibacterial activity comes mainly from the silver and not from PES [23,51,52].

In general, the inhibition zones reported by different authors are larger than the ones observed in this work, which can be explained to by the hypothesis that the release of silver nanoparticles is amplifying the biocidal zone. Once the nanoparticles are not well attached to the membranes, they can diffuse in the media and inhibit bacterial growth. However, in this current work, the silver release is reduced to a small region, corroborating this hypothesis. Thus, the sputtering technique was efficient at entrapping silver nanoparticles on the membrane, and their biocidal effect will be concentrated in this region, which may be noteworthy to inhibit biofouling formation.

#### 3.3.2. The Biofouling Resistance Test

**Figure 8:**
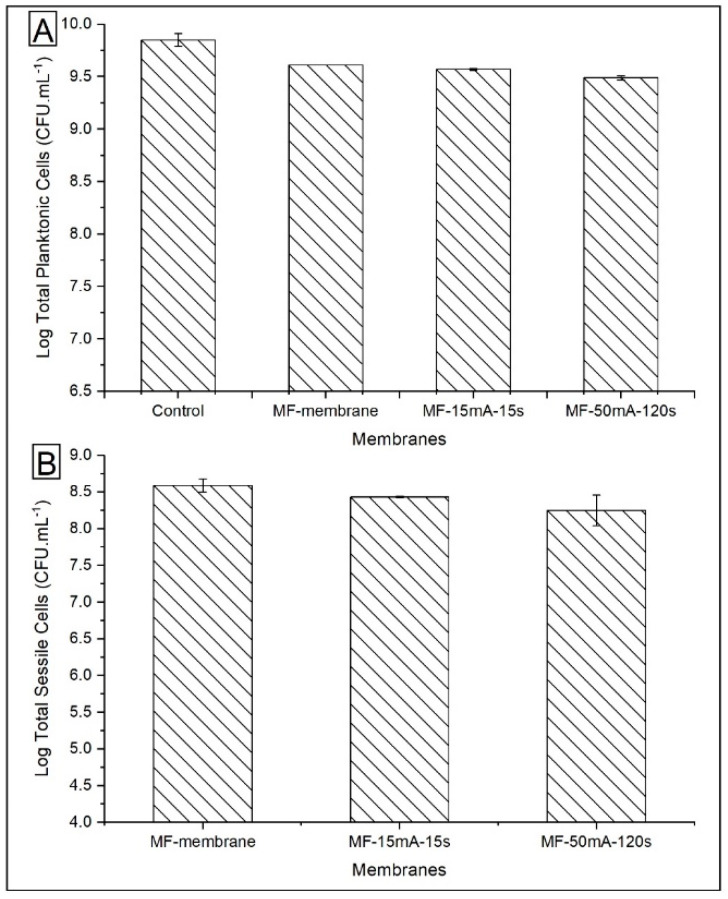
Total cells of *P. aeruginosa* after their exposure to MF membrane and MF-AgNps: (**A**) total planktonic cells and (**B**) total sessile cells. Number of bacteria initially inoculated on each sample: log CFU mL^−1^ = 7.0 at 30 °C, 200 rpm for 24 h of incubation.

The quantification of total planktonic cells and total sessile cells, shown in Figure 8, did not indicate any significant difference between the membranes, with the CFU mL^−1^ being in the same order of magnitude for all membranes.

**Figure 9:**
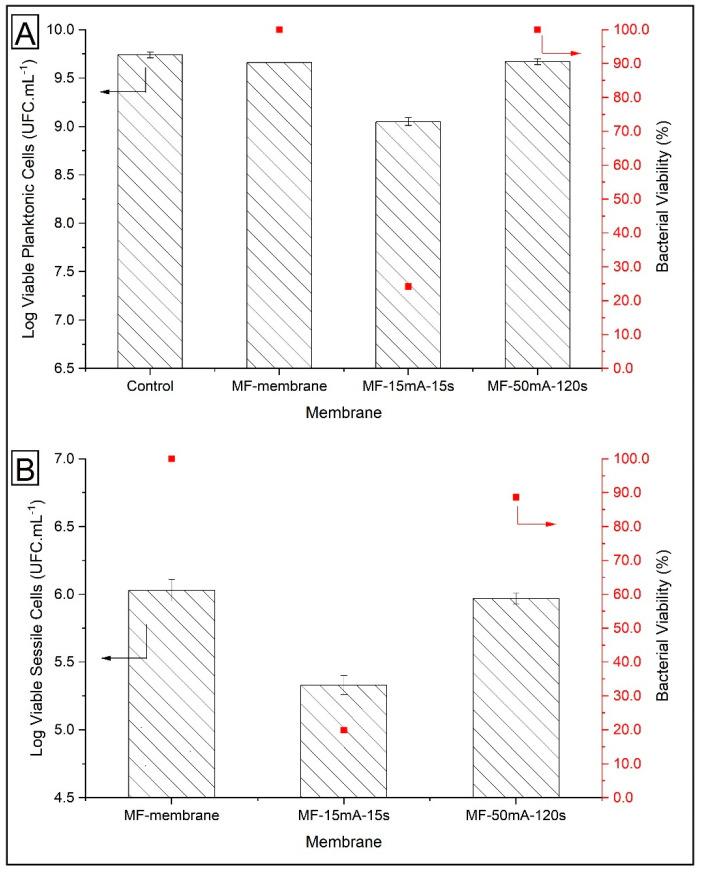
*P. aeruginosa* cells viabilities after their exposure to MF-membrane and MF-AgNps: (**A**) viable planktonic cells and (**B**) viable sessile cells. Number of bacteria initially inoculated on each sample: log CFU mL^−1^ = 7.0 at 30 °C, 200 rpm for 24 h of incubation.

The quantification of viable planktonic cells grown in suspensions without membrane (control sample) and with MF membrane (without AgNps) did not show a significant difference (Figure 9). On the other hand, for MF-15mA-15s, there was a considerable reduction of ~0.6 log units in CFU mL^−1^ compared to MF-membrane. In this case, after the exposure to MF-AgNps membranes for 24 h, the viability of the planktonic cells was only 24.2%. This decrease demonstrated that the MF-15mA-15s membrane causes an expressive *P. aeruginosa* growth inhibition.

Figure 9 also depicts the viable sessile cells on the different membranes. A comparison between MF membrane and MF-15mA-15s indicates that the bacterial growth showed a 0.7 log unit reduction in CFU mL^−1^, while the viability of the sessile cells was 19.8% after 24 h of exposure to AgNps. This result confirmed that the silver impregnated in MF-15mA-15s improved the bactericidal properties of the membranes, which is in agreement with other studies that investigated silver nanoparticles synthesized by chemical route [25,51].

The adhesion of *P. aeruginosa* in the MF membrane and MF-50mA-120s was investigated during a cross-flow experiment in order to evaluate their performance in a filtration process. The results revealed 0.66 ± 0.02 log unit reduction of viable sessile cells, and as a consequence, 22% of bacterial viability for MF-50mA-120s (log MF-membrane = 6.63, log MF-50mA-120s = 5.97). These results show an outstanding maintenance of the effectiveness of the MF-50mA-120s in comparison to the MF membrane, even with a continuous flowrate through the membrane. 

Liu et al. (2013) [53] observed the deposition of *E. coli* in polysulfone membranes impregnated with silver nanoparticles synthesized by chemical route. The authors concluded that the AgNps did not affect the kinetics of bacterial deposition. However, the bacterial detachment ratio during rising is large in the presence of silver nanoparticles because the bacteria become inactivated after the contact with these nanoparticles, enhancing the detachment rate. 

Thereby, in this study, the adhesion of *P. aeruginosa* in MF-AgNps was verified and quantified as the total sessile cells. Nevertheless, the viable sessile cells decreased in comparison with the MF membrane, indicating that AgNps inactivated the microorganisms, which would facilitate the detachment. 

Even though the exact mechanism of antibacterial activity of AgNps is not fully understood and further studies are needed to explain this gap, several researchers agree on a synergistic action between contact killing of nanoparticles and the releasing of silver ions from the membranes [15,54,55].

In this context, the antibacterial effect of MF-AgNps membranes produced in this work is affected by its lower concentration of silver leached; however, for applications in water treatment, for example, it is important to minimize this leaching due to the concern with human health and environment protection. Furthermore, the development of membranes with biocidal advantages associated with a long lifespan is needed. 

Therefore, the MF-AgNps membrane demonstrated potential for these applications because of its antibacterial properties and its expected longer lifespan due to its low silver leaching. 

## 4. Conclusions

Impregnation of silver nanoparticles on the microfiltration membranes by sputtering technique was confirmed by different and complementary analyses. From FESEM images, average diameters of 88 and 50 nm were estimated for MF-15mA-15s and MF-50mA-120s, respectively, suggesting that the increase in sputtering time and sputtering current reduces the diameter of silver particles. The diffractogram of the MF-50mA-120s membrane exhibited a sharp peak at 2 *θ* = 38°, which is attributed to the crystallinity of silver, proving the presence of silver on the surface of this membrane and corroborating the FESEM and EDS observations.

One of the main advantages of the sputtering technique is the good adhesion between AgNps and membranes, which allows the release of silver ions lower than the maximum limit of silver in drinking water by World Health Organization and the U.S. Environmental Protection Agency. After 10 months of immersion, there was still approximately 93.0 and 87.9% of silver initially impregnated onto MF-15mA-15s and MF-50mA-120s membranes, respectively. Furthermore, after 24 h of filtration test in full-recycle set-up with high flowrate, MF-50mA-120s showed that 98.8% of silver remained on the membrane. These results indicate the efficiency of sputtering technique to entrapped silver nanoparticles on the membranes. 

The disc diffusion method demonstrated the antibacterial activity of silver nanoparticles impregnated onto membranes by an inhibition zone of approximately 0.5 and 0.8 mm for MF-15mA-15s and MF-50mA-120s, respectively. The microbial inhibition occurs with the diffusion of silver nanoparticles from the membrane into the agar layer. Thereby, these small inhibition zones can be explained by the lower release of silver nanoparticles impregnated on the microfiltration membranes by sputtering method. 

The biofouling resistance test for MF-15mA-15s showed 0.6 and 0.7 log unit reductions in CFU mL⁻^1^ when compared with the MF membrane (without AgNps) for viable planktonic cells and viable sessile cells, respectively. After exposure to this membrane for 24 h, the planktonic cells and sessile cells viabilities were both under 25%, confirming the biocidal property of the membrane with silver nanoparticles.

Although there was adhesion of *P. aeruginosa* in microfiltration membranes impregnated with AgNps, the viable sessile cells decreased, indicating a great potential of silver nanoparticles to reduce biofouling and its consequences.

The highlight of this work is the occurrence of bacterial inhibition in membranes impregnated with AgNps with reduced silver leaching, which allows a longer lifespan for these membranes. Less silver loss was observed in this work, even though it was analyzed in long-term immersions and in filtration experiments with higher flowrate when compared with other studies in the literature.

## Figures and Tables

**Table 1:** Water permeability of MF membrane and membranes loaded with AgNps.

Membrane	Water Permeability (L h^−1^ m^−2^ bar^−1^)	Rejection (%)
MF-membrane	6349.9 ± 475.1	26.4
MF-15mA-15s	6455.0 ± 519.9	-
MF-50mA-120s	6388.0 ± 564.8	78.3

## Supplementary Materials

The following are available online at https://www.mdpi.com/2073-4360/12/8/1686/s1, Figure S1: FTIR spectra of MF-membrane, MF-15mA-15s and MF-50mA-120s.
