# Supplementary material for: Investigation of Biocidal Effect of Microfiltration Membranes Impregnated with Silver Nanoparticles by Sputtering Technique
Source: Polymers (Basel). 2020 Jul 29;12(8):1686. doi: 10.3390/polym12081686 (PMC7463648; doi:10.3390/polym12081686)
Supplement: Supplementary file 1 [file polymers-12-01686-s001.pdf]

polymers

MDPI

Article

Investigation of Biocidal Effect of Microfiltration Membranes Impregnated with Silver Nanoparticles by Sputtering Technique

Aline M.F. Linhares [1,*], Cristiano P. Borges [2] and Fabiana V. Fonseca [1]

[1] School of Chemistry, Federal University of Rio de Janeiro, Horacio Macedo Av, 2030, Technology Center, I-124, University City, Rio de Janeiro, RJ 21941-909, Brazil; fabiana@eq.ufrj.br
[2] Chemical Engineering Program, COPPE, Federal University of Rio de Janeiro, Horacio Macedo Av, 2030, Technology Center, G-115, University City, Rio de Janeiro, RJ 21941-450, Brazil; cristiano@peq.coppe.ufrj.br
* Correspondence: alinemarquesrj@hotmail.com

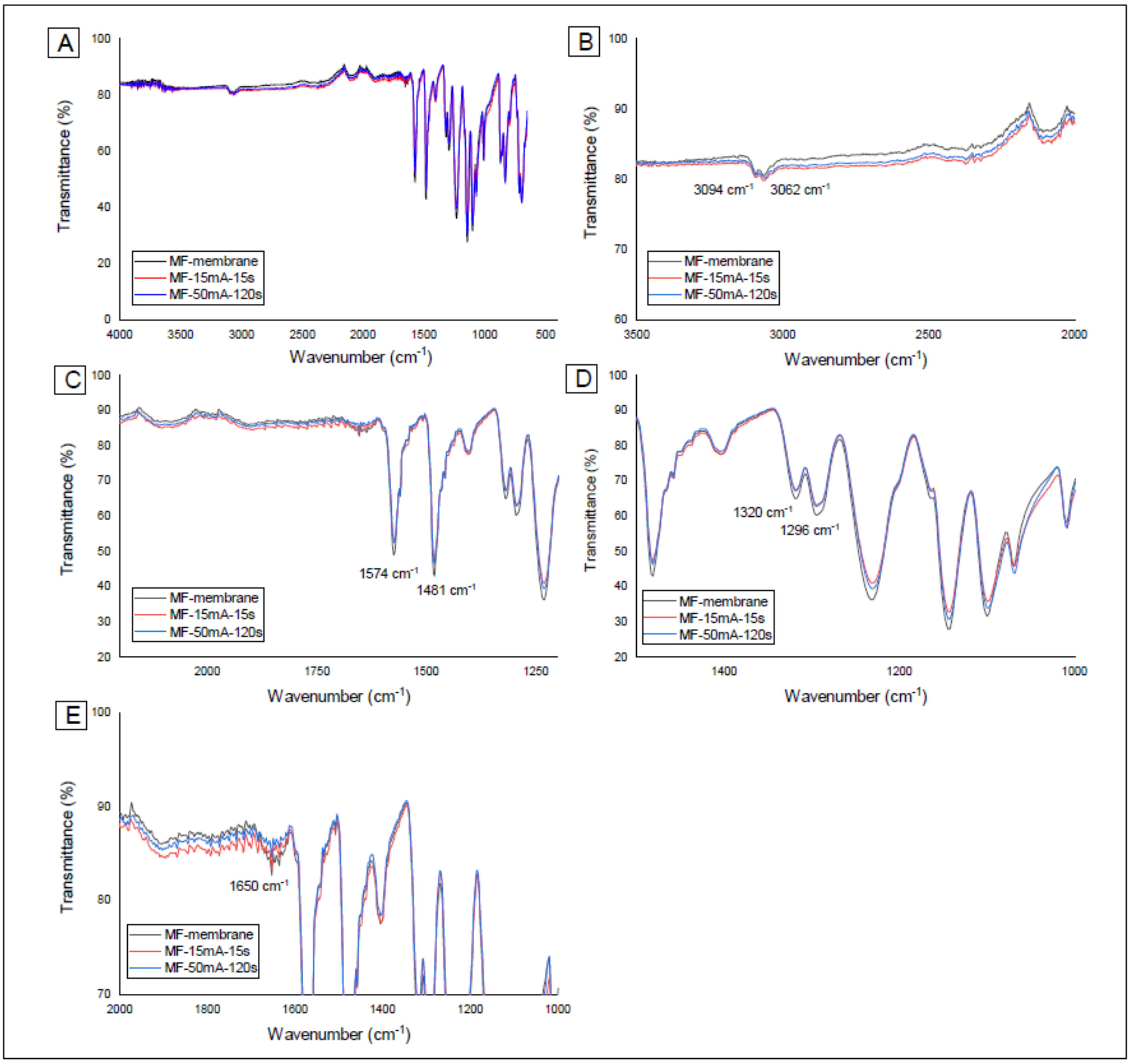

Figure S1. FTIR spectra of MF-membrane, MF-15mA-15s and MF-50mA-120s.
